# Colorimetric Visualization Using Polymeric Core–Shell Nanoparticles: Enhanced Sensitivity for Formaldehyde Gas Sensors

**DOI:** 10.3390/polym12050998

**Published:** 2020-04-25

**Authors:** Jae Jung Park, Yongsoo Kim, Chanmin Lee, Jun-Won Kook, Donghyun Kim, Jung-Hyun Kim, Ki-Seob Hwang, Jun-Young Lee

**Affiliations:** 1 Department of Chemical and Biomolecular Engineering, Yonsei University Yonsei-ro 50, Seodaemun-gu, Seoul 120-749, Korea; qkrwownd11@kitech.re.kr (J.J.P.); ratian36@kitech.re.kr (D.K.); jayhkim@yonsei.ac.kr (J.-H.K.); 2 Research Institute of Sustainable Manufacturing System, Intelligent Sustainable Materials R&D Group, Korea Institute of Industrial Technology, 89 Yangdaegiro-gil, Ipjang-myeon, Seobuk-gu, Cheonan-si, Chungcheongnam-do 31056, Korea; bohemian4215@kitech.re.kr (Y.K.); clee@kitech.re.kr (C.L.); kukjw83@kitech.re.kr (J.-W.K.); 3 Department of Polymer Science & Engineering, Sung Kyun Kwan University 2066, Seobu-ro, Jangan-gu, Suwon-si, Gyeonggi-do 16419, Korea

**Keywords:** core–shell nanoparticle, colorimetric sensor, formaldehyde, polyethyleneimine, pH indicator

## Abstract

Although equipment-based gas sensor systems (e.g., high-performance liquid chromatography) have been widely applied for formaldehyde gas detection, pre-treatment and expensive instrumentation are required. To overcome these disadvantages, we developed a colorimetric sensor based on polymer-based core–shell nanoparticles (PCSNPs), which are inexpensive, stable, and exhibit enhanced selectivity. Spherical and uniform poly(styrene-co-maleic anhydride) (PSMA)/polyethyleneimine (PEI) core–shell nanoparticles were prepared and then impregnated with Methyl Red (MR), Bromocresol Purple (BCP), or 4-nitrophenol (4-NP) to construct colorimetric sensors for formaldehyde gas. The intrinsic properties of these dyes were maintained when introduced into the PCSNPs. In the presence of formaldehyde, the MR, BCP, and 4-NP colorimetric sensors changed to yellow, red, and gray, respectively. The colorimetric response was maximized at a PEI/PSMA ratio of four, likely owing to the high content of amine groups. Effective formaldehyde gas detection was achieved at a relative humidity of 30% using the MR colorimetric sensor, which exhibited a large color change (92%) in 1 min. Advantageously, this stable sensor allowed sensitive and rapid naked-eye detection of low formaldehyde concentrations (0.5 ppm). Hence, this approach is promising for real-time formaldehyde gas visualization and can also be adapted to other colorimetric gas sensor systems to improve sensitivity and simplicity.

## 1. Introduction

Colorimetric sensors are considered as potentially efficient detection methods for environmental monitoring of harmful gases, such as SO_x_, NO_x_, formaldehyde, and CO_2_. Notably, formaldehyde gas exposure can cause serious reproductive system diseases, as well as bronchial cancer and leukemia [1,2,3,4,5]. The World Health Organization (WHO) has established a safe-exposure standard of 80 ppb averaged over 30 min. In addition, the Occupational Safety and Health Administration (OSHA) has set the permissible exposure limit (PEL) at 750 ppb and the immediately dangerous to life or health (IDLH) limit at 20 ppm [6,7,8,9]. Thus, the development of formaldehyde sensor systems has been studied for applications in a variety of areas such as clinical analysis, environmental monitoring, biotechnology, and the pharmaceutical and food industries [10,11,12,13].

Current systems for formaldehyde gas detection are typically based on high-performance liquid chromatography (HPLC), ion chromatography (IC), voltammetry, amperometry, enzyme electrodes, and solid-state sensors. However, equipment-based systems (e.g., HPLC and IC) typically require pre-treatment processes and expensive instrumentation, which complicates the detection of formaldehyde gas in real time [14,15,16,17,18].

To overcome these disadvantages, researchers have suggested colorimetric pH sensors based on a solid matrix (e.g., polymer and sol–gel materials) and nanostructured particles. Solid matrices impart selectivity and provide permeability, flexibility, and mechanical and chemical stability. There are various methods, including covalent binding, entrapment, and adsorption, available for the immobilization of pH indicators in solid matrices. However, immobilization by covalent binding requires the presence of appropriate functional groups and both the adsorption and entrapment methods are limited by leaching of the indicator dye [19]. For this reason, various nanostructured particles (e.g., organic, inorganic, and hybrid materials) have been suggested as potential colorimetric pH sensors because of their high sensitivities, large surfaces, large volume ratios, and high specific surface areas [20,21,22,23,24,25]. Recently, formaldehyde sensors based on various nanostructures, including nanoflower and nanosheet structures, have been suggested. Notably, an In_2_O_3_/ZnO nanoflower sensor showed good sensitivity at a formaldehyde concentration of 5 ppm. Furthermore, Zhang et al. reported that a sensor fabricated from graphene/ZnO composite nanosheets exhibited a response at a formaldehyde concentration of 25 ppm at room temperature [21]. Nevertheless, various obstacles have severely restricted the application of nanoparticles in sensors. For instance, expensive metallic materials are cytotoxic because certain metal ions can be formed upon dissolution [26].

Herein, we suggest polymer-based core–shell nanoparticles (PCSNPs) incorporating an optical pH indicator mechanism as an efficient colorimetric sensor that can be used to visualize formaldehyde gas [27]. Generally, the formaldehyde sensor mechanism involves a nucleophilic addition reaction between formaldehyde and an amine group to form an imine (Schiff base) [28]. The pH shift that accompanies the formation of a Schiff base can be exploited to generate a color change using pH indicator dyes [29]. A PCSNP with a shell of polyethyleneimine (PEI) is expected to have numerous sites for Schiff base creation under formaldehyde conditions. In this system, poly(styrene-co-maleic anhydride) (PSMA) was selected as the core material and ion transporter because of the high reactivity between maleic anhydride (MA) and PEI [30,31]. Commonly, optical pH sensors employ pH indicator dyes, which are typically weak organic acids or bases with distinct optical properties associated with their protonated (acidic) and deprotonated (basic) forms [20,32,33,34]. Thus, we utilized indicator dyes with a variety of pH ranges to achieve Schiff base visualization.

In this study, to develop a colorimetric sensor for formaldehyde gas detection, PSMA/PEI core–shell nanoparticles were prepared. Subsequently, the hydrophobic sites of PSMA were impregnated with Bromocresol Purple (BCP), Methyl Red (MR), or 4-nitrophenol (4-NP). To verify the effect of formaldehyde on the colorimetric response of the fabricated sensors, we determined the detection limits and analyzed the effects of the PEI/PSMA ratio, humidity, and exposure time. The current work describes, for the first time, a core–shell-type PSMA/PEI sensor that acts as an efficient formaldehyde gas detection system that can be used as the basis for developing a colorimetric gas sensor.

## 2. Materials and Methods

### 2.1. Materials

All chemical reagents used in this study were general purpose reagents and deionized water was used to prepare all solutions. Styrene (99% purity), MA, PEI (branched, average Mw 25,000), MR, BCP, and 4-NP were purchased from Sigma-Aldrich, Inc. (St. Louis, MO, USA). The initiator, potassium persulfate (KPS), was provided by Daejung Chemicals and Metals Co. Ltd. (Siheung, Korea). A gas mixture consisting of 20 µmol formaldehyde/mol nitrogen was obtained from RIGAS (Daejeon, Korea).

### 2.2. Preparation of Core–Shell-Type Formaldehyde Sensors

Prior to the preparation of PSMA, the polymerization inhibitor of styrene was removed using ammonium oxide. Then, using a 250-mL double-jacketed reactor equipped with a cooling condenser, a circulation pump, and a mechanical stirrer, the soap-free emulsion polymerization of PSMA was performed under a nitrogen atmosphere, as follows. MA (1.5 g, 15 wt%) in 155 mL of purified distilled water was placed in the prepared reactor and stirred for 10 min. Then, purified styrene (8.5 g, 85 wt%) was added to the MA dispersion in the reactor and stirred for 30 min. After 30 min, KPS (0.17 g, 0.2 wt%), which is a water-soluble initiator, was dissolved in 5 g of distilled water and injected at a rate of 0.5 mL/min over 10 min using a syringe pump (NE 300). After the addition of the initiator, the polymerization was performed for 5 h at 300 rpm and 70 °C. Subsequently, the temperature was rapidly lowered to 30 °C and PEI (Mw 25,000) was added. The ratio of added PEI varied from 1 to 10 relative to the weight of MA introduced into PSMA. Specifically, a PEI solution was prepared by adding the required amount of PEI to 20 mL of distilled water and allowing it to dissolve for 30 min. Then, 10 mL of the prepared PEI solution was added to 10 mL of the PSMA solution and the mixture was stirred at 150 rpm for 24 h to prepare PSMA/PEI core–shell nanoparticles.

Finally, the PSMA/PEI core–shell nanoparticles were impregnated with a pH indicator dye. The dye solutions were prepared by dissolving 0.4 g of dye (MR, BCP, or 4-NP) in 20 mL of dichloromethane. Then, 0.4 µL of a dye solution was added to 5 mL of the PSMA/PEI core–shell nanoparticles and the mixture was stirred for 3 h to form a formaldehyde sensor. The sensor particles were then washed three times with deionized water to remove any residual dye, filtered, and dried for 12 h in a vacuum oven to form a formaldehyde sensor powder.

### 2.3. Morphological Analysis of PSMA/PEI Core–Shell Nanoparticles

Fourier transform infrared (FT-IR) spectroscopy (Nicolet 6700) was used to confirm the reaction between PSMA and PEI. The FT-IR samples were prepared by drying PSMA and PSMA/PEI in a vacuum oven at 30 °C for 12 h. The structure of the polymer was confirmed by solid-state ^13^C-nuclear magnetic resonance (NMR) spectroscopy (Bruker, Avance II, 500 MHz). In addition, ^1^H-NMR analyses were performed to confirm the interaction between PEI and formaldehyde as well as the effects of the dye. Ultraviolet-visible (UV-Vis) spectroscopy (Jasco V-730) was used to verify the impregnation of the dyes in PSMA/PEI. The samples for the UV-Vis measurements were prepared by dissolving 0.1 g of the formaldehyde sensors or the pH dyes in *N*-methyl-2-pyrrolidone. Scanning electron microscopy (SEM; JEOL JSM-6700) was used to observe the morphologies of the synthesized nanoparticles. The SEM samples were prepared by dropping the synthesized nanoparticle dispersion on a silicon wafer and drying at room temperature for 10 h. The core–shell structure of the nanoparticles was observed by transmission electron microscopy (TEM; JEM-F200). The TEM sample was prepared by placing 1–2 drops of the PSMA/PEI nanoparticle dispersion on a copper grid and drying at room temperature for 12 h. In addition, the carbon and nitrogen contents were analyzed by energy-dispersive X-ray spectroscopy (EDS) to determine the distribution of PEI in the PSMA/PEI core–shell nanoparticles. Dynamic light scattering (DLS; Zetasizer nano ZS90) was used to analyze the stability of the nanoparticles and observe the effect of the PEI content on the particle size. The DLS samples were prepared by adding 1 wt% of the nanoparticle dispersion to distilled water.

### 2.4. Gas Sensor Measurements

A gas permeation apparatus was manufactured to observe the color changes resulting from formaldehyde detection. Formaldehyde gas, dry nitrogen gas, and wet nitrogen gas were used as gas sources for the gas permeation device, and the total flow rate (up to 1000 sccm) was controlled using multigas flow controllers (MFCs). The flow rate of the source was continuously adjusted to control the formaldehyde concentration in the gas flowing into the chamber. Nitrogen (wet) was used to control the humidity and temperature, which are external conditions. The temperature effect was investigated by increasing the temperature in steps of 5 °C from 30 to 50 °C. The effect of humidity was examined by adjusting the humidity in steps of 10% from 5% to 90%. The chamber connected to the gas line contained a hygrometer and a thermometer. In addition, the bottom of the chamber was transparent so that color changes could be observed using a scanner (HP Scanjet Pro 3500). The sample was prepared by dropping the dispersion of PSMA/PEI particles loaded with dye onto filter paper (6 × 6 cm) and drying. It was dried at room temperature for 30 min in a nitrogen atmosphere. The performance of the formaldehyde gas sensors with the various dyes was investigated by exposing the sensors to different gas concentrations and humidities for 0–5 min, and the resulting color changes were expressed as ΔRGB values. The measured results are expressed as the average of five repeated measurements.

## 3. Results and Discussion

### 3.1. Effect of PEI Content on the Structural Properties of PSMA/PEI Core–Shell Nanoparticles

In this study, a formaldehyde detector was developed based on PSMA/PEI core–shell nanoparticles doped with a pH indicator dye that undergoes a pH shift in the presence of formaldehyde gas.

**Scheme 1:**
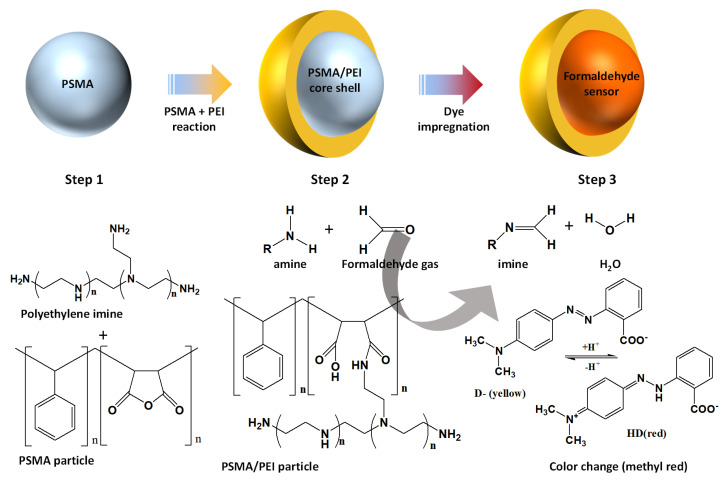
Schematic illustration of the preparation of a colorimetric sensor for formaldehyde gas based on poly(styrene-co-maleic anhydride) (PSMA)/polyethyleneimine (PEI) core–shell nanoparticles doped with a pH indicator dye.

Binding between formaldehyde gas and the PEI shell is expected to result in a change in the interparticle color. Specially, nucleophilic addition of the aldehyde of formaldehyde to an amine of PEI forms an imine. The associated pH shift causes the pH dye embedded in the PSMA core to transform from the base form to the acid form, which results in a color change. The process used to prepare the PSMA/PEI core–shell nanoparticles in which the core is doped with a pH indicator dye (BCP, MR, or 4-NP) is illustrated in Scheme 1. PSMA particles were formed by the radical copolymerization of styrene and MA. Then, a shell layer was formed via a ring-opening reaction between a carboxyl group in MA and an amine group in PEI. Subsequently, the core was impregnated with a hydrophobic pH dye.

**Figure 1:**
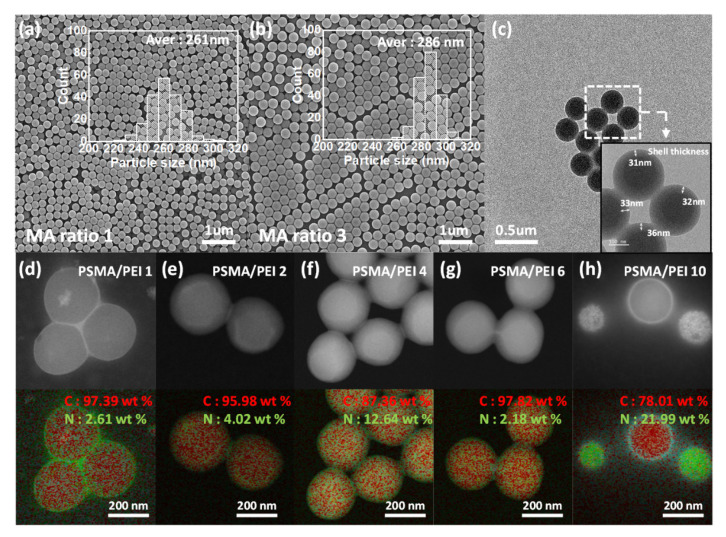
(**a**,**b**) SEM images of PSMA nanoparticles prepared with different maleic anhydride (MA) ratios; (**c**) TEM image of PSMA/PEI core–shell nanoparticles; (**d**) EDS mapping of the carbon and nitrogen contents in the shells of PSMA/PEI core–shell nanoparticles prepared with various PEI/PSMA ratios: (**d**) 1, (**e**) 2, (**f**) 4, (**g**) 6, and (**h**) 10.

The TEM and SEM images (Figure 1a–c) show that the prepared PSMA and PSMA/PEI core–shell nanoparticles are spherical, uniform, and well dispersed. Furthermore, the SEM images show that the PSMA particle size tends to increase slightly as the MA ratio increases (Figure 1a,b). When the MA ratio was increased from 1 to 3, the average particle size distribution increased to approximately 25 nm. This change is due to the surfactant effect of MA in the soap-free emulsion system. When PEI is added to the PSMA nanoparticles, the particle size increases slightly from 220 nm to 260 nm up to a PEI/PSMA ratio of four. Although the particle size increased further at a PEI/PSMA ratio of six, the volume (v/v%) decreased, indicating that the particle size was nonuniform owing to the aggregation of secondary PEI particles.

To further explain the above-mentioned phenomenon, when PEI is added to the PSMA nanoparticles, the particle size increases up to a certain ratio. However, the addition of PEI changes the surface energy relative to the force required for reaction between the –OH groups present in the PSMA nanoparticles. Thus, reactions between PEI molecules become more favorable and secondary particles are formed, as confirmed by the TEM and EDS data in Figure 1h.

Therefore, the correlation between the PEI/PSMA ratio and the particle size was investigated using DLS and EDS (Figure 1d–h, Figure S1 and Table S1), which revealed that the particle size was not related to the PEI content. Moreover, the EDS analysis showed that the highest nitrogen content was achieved at a PEI/PSMA ratio of four (Figure 1f) (based on the N mapping, as shown in Figure 1d–h). In addition, secondary particles were only observed at a PEI/PSMA ratio of 10. It is likely that a PEI/PSMA ratio of four is ideal for the reaction with MA moieties at the interface, resulting in the formation of a core–shell structure. However, the presence of excess PEI results in the formation of secondary particles. Therefore, at a PEI/PSMA ratio of four, a large amount of amine groups are present, which is expected to enhance the efficacy of the colorimetric sensor.

To investigate the impregnation of the pH dyes in the PSMA core, a UV-Vis spectroscopy analysis was conducted. As shown in Figure S2, the formaldehyde sensors with BCP, MR, and 4-NP contained 2751, 2650, and 692 ppm of the corresponding pH dyes, respectively. This result implies that the contents of the pH dyes were related to their hydrophilicities in the emulsion state.

### 3.2. Polymerization Reaction

**Figure 2:**
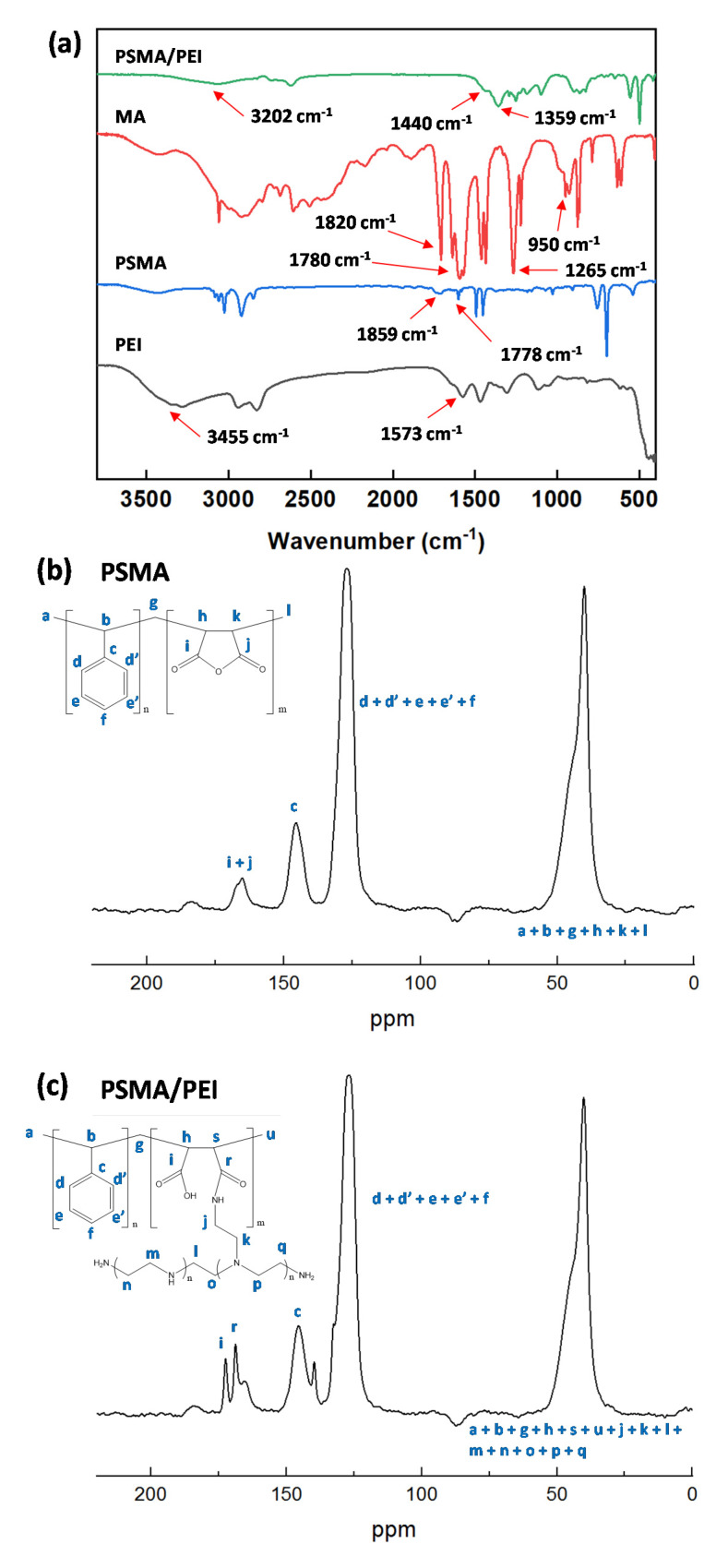
(**a**) FT-IR spectra of PSMA/PEI core–shell nanoparticles, PSMA, PEI, and MA; ^13^C-NMR spectra (500 MHz) of (**b**) PSMA and (**c**) PSMA/PEI.

The successful polymerization of the PSMA (92% conversion) and PSMA/PEI core–shell nanoparticles was confirmed using FT-IR spectroscopy and solid-state ^13^C-NMR spectroscopy (Figure 2). In the FT-IR spectrum of PSMA (Figure 2a), the bands at 1859 and 1778 cm^−1^ were assigned to the carbonyl groups of the MA moieties. Furthermore, a band appeared at 1695 cm^−1^, which corresponded to carboxyl groups. In contrast, the FT-IR spectrum of the core–shell nanoparticles exhibited amide peaks at 1440 and 1359 cm^−1^, which are related to the aminolysis reaction [35]. As shown in Figure 2a, a decrease in the carbonyl stretching peak at 1778 cm^−1^ occurred for the PSMA/PEI core–shell nanoparticles, while the amine stretching peak at 1440 cm^−1^ increased. Furthermore, for the PSMA/PEI core–shell nanoparticles, new strong bands were observed at 1359, 1440, and 3202 cm^−1^, which were attributed to N–H vibrations. This result confirms the formation of amide groups (–CONH–). Additionally, the decrease in the C–O stretching band at 1265 cm^−1^ confirms that a ring-opening reaction occurred between an amine (PEI) and a carboxyl group (MA). These observations clearly indicate that PEI was successfully grafted to PSMA.

The solid-state ^13^C-NMR results (Figure 2b,c) further confirm the ring-opening reaction between an amine and a carboxyl group. In the PSMA particles, the methylene and tertiary carbons of the PSMA main chain were observed at 28–49 ppm, as shown in Figure 2b. Furthermore, strong signals were observed at 117–128 ppm, which correspond to the methylene group by the terminal cumene. In contrast, for the PSMA/PEI core–shell nanoparticles, a new bimodal signal was observed at 163–172 ppm, which corresponds to the ring-opening reaction between an amine group and a carboxyl group. Therefore, the solid-state ^13^C-NMR results are consistent with the FT-IR spectra and explicitly show that the polymerization of the PSMA/PEI core–shell nanoparticles was successful.

Additionally, the occurrence of a chemical reaction between the PSMA/PEI core–shell nanoparticles and formaldehyde was confirmed by examining the reaction between PEI and formaldehyde in the presence of MR using ^1^H-NMR spectroscopy (Figure S3). In the ^1^H-NMR spectrum of the PEI + formaldehyde sample, a new signal was observed at 8.43 ppm, which corresponds to the formation of an imine [36]. Thus, the ^1^H-NMR results confirm that a nucleophilic addition reaction occurs between an amine in PEI and formaldehyde.

### 3.3. Sensitivity of Colorimetric Sensors to Formaldehyde

**Scheme 2:**
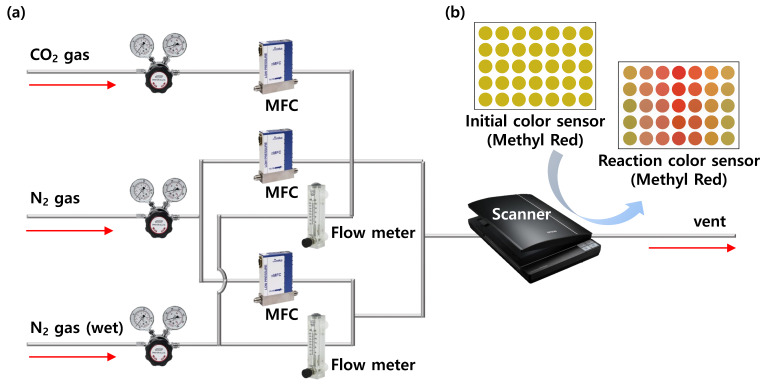
Schematic illustration of the sensor test system with multi-gas flow controllers (MFCs). (**a**) MFC system. The flow rate of the source was continuously adjusted to control the formaldehyde concentration in the gas flowing into the chamber (up to 1000 sccm). (**b**) Sensor test system. The bottom of the chamber was transparent so that color changes could be observed using a scanner.

**Figure 3:**
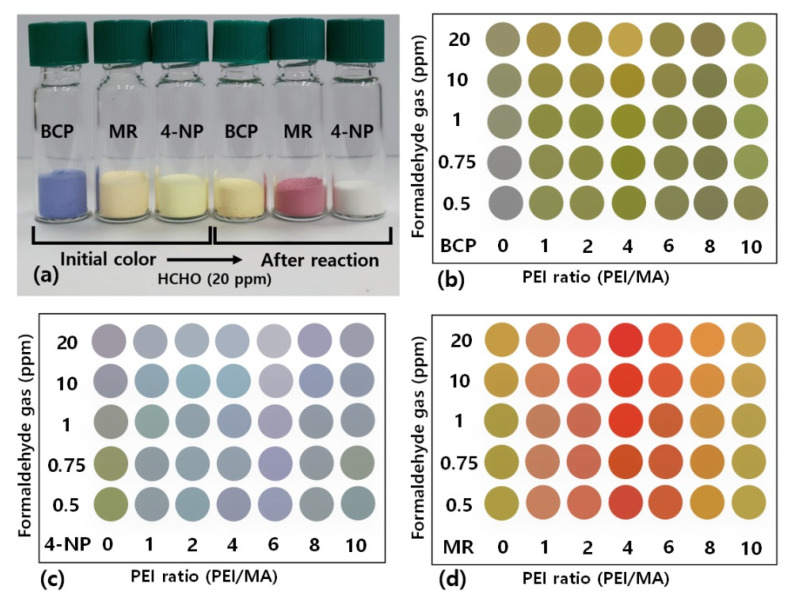
(**a**) Color changes observed by the naked eye for the bromocresol purple (BCP), 4-nitrophenol (4-NP), and methyl red (MR) colorimetric sensors when exposed to formaldehyde gas (20 ppm). Colorimetric responses (ΔRGB values) of (**b**) BCP, (**c**) 4-NP, and (**d**) MR sensors prepared with various PEI/PSMA ratios (0–10) on exposure to 20 ppm formaldehyde gas.

The colorimetric sensor performance was evaluated using a sensor test system with MFCs (Scheme 2). As shown in Figure 3, when the colorimetric sensors incorporating various dyes (BCP, 4-NP, or MR) and PEI ratios were exposed to formaldehyde gas, color changes were observed.

As shown in Figure 3a, the various sensors exhibited different colors after exposure to 20 ppm of formaldehyde gas. The colorimetric sensors were blue, light yellow, and yellow before gas exposure (left), but turned yellow, white, and red, respectively, after the gas exposure (right). However, the formaldehyde gas sensors did not exhibit color changes at room temperature and humidity. Figure 3b–d show the degree of color change based on the PEI content and formaldehyde gas concentration. In particular, the color change of the MR sensor (Figure 3d) is more prominent than those of the BCP and 4-NP sensors (Figure 3b,c). Notably, the initial colors of the dyes were retained within the core–shell nanoparticles. This result implies that PCSNPs are suitable as colorimetric gas sensors.

**Figure 4:**
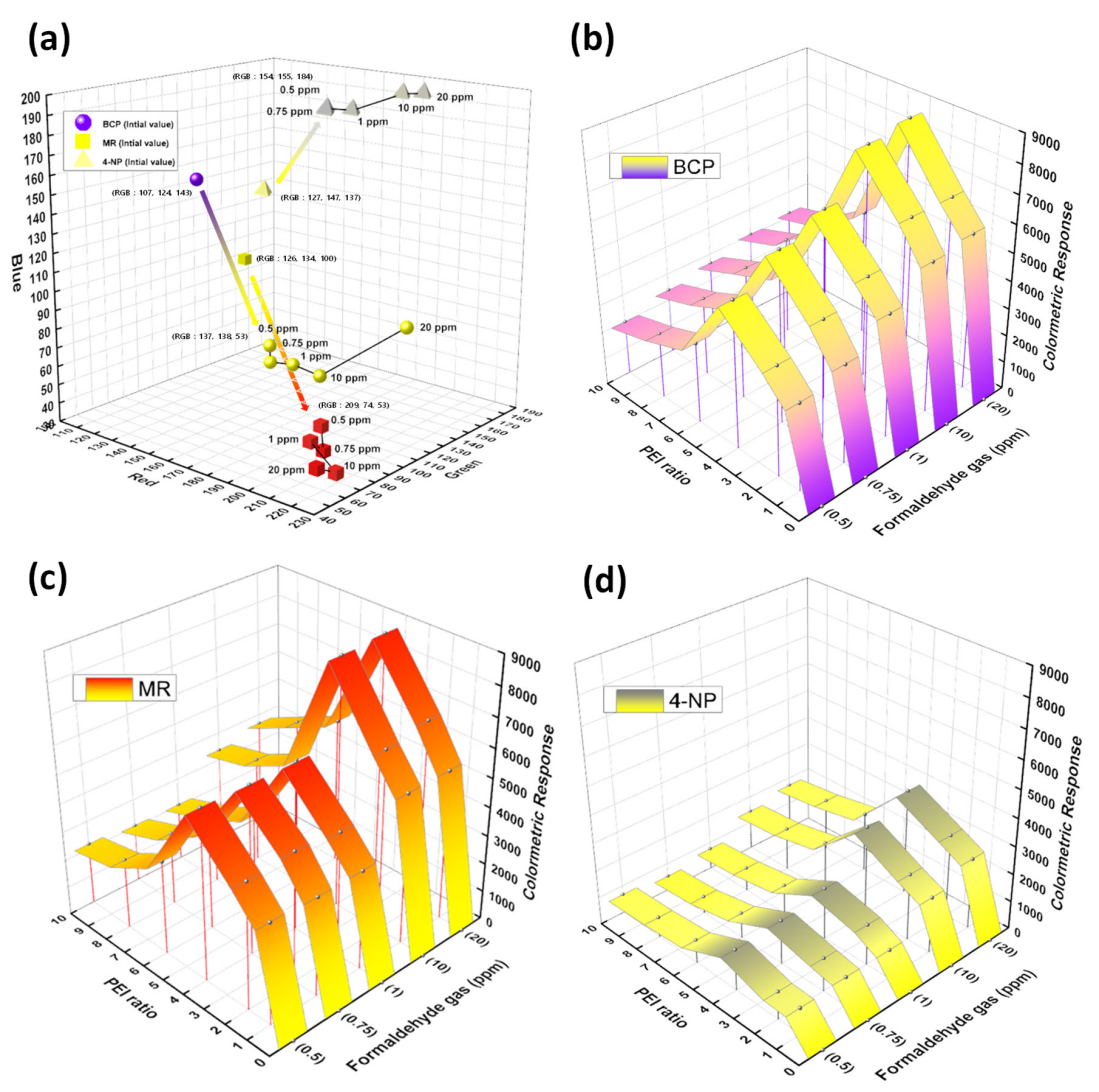
(**a**) Color changes observed for BCP, MR, and 4-NP colorimetric sensors when exposed to 0.5–20 ppm formaldehyde. Colorimetric response (∆RGB values) of (**b**) BCP, (**c**) MR, and (**d**) 4-NP sensors prepared with various PEI/PSMA ratios (0–10) on exposure to 0.5–20 ppm formaldehyde.

The effects of various factors (e.g., formaldehyde gas volume, PEI/PSMA ratio, dye, response time, and humidity (RH%)) on the color shift performance of the colorimetric sensors were evaluated using a sensor test system with MFCs. Although digital MFCs are commonly used to evaluate the color change performance under formaldehyde gas conditions, we added a temperature controller to the MFCs to prevent temperature-induced color transitions. As shown in Figure 4a, the initial RGB values of the formaldehyde sensors with BCP, MR, and 4-NP were (107, 124, 143), (126, 134, 100), and (117, 147, 137), respectively. Following exposure to formaldehyde gas, the colors of the BCP, MR, and 4-NP colorimetric sensors were observed to change to yellow, red, and gray, respectively (Figure 4a). To visualize the colorimetric response of the sensors, the ΔRGB values were calculated [i.e., (ΔR^2^ + ΔG^2^ + ΔB^2^)^1/2^].

The colorimetric responses (ΔRGB values) of the BCP, MR, and 4-NP sensors, with respect to the PEI/PSMA ratio and formaldehyde concentration, are summarized in Table 1, Tables S2 and S3, Figure 4b–d, and Figure S4. Table 1 shows the dependence of the ΔRGB values on the PEI content at a formaldehyde concentration of 20 ppm. A color change was observed from a PEI/PSMA ratio of one, with the highest color change response observed at a PEI/PSMA ratio of four, which was considered the 100% response. At PEI/PSMA ratios of one, two, and four, the color change responses of the BCP sensor were 66%, 76%, and 100%, respectively. As the PEI content increased further, the color change response decreased up to 36%. The same trend was observed for the MR and 4-NP sensors.A colorimetric response was observed under all the formaldehyde conditions, even at the lowest formaldehyde concentration of 0.5 ppm. The colorimetric response increased as the PEI/PSMA ratio increased from 1 to 4, but then rapidly decreased from a PEI/PSMA ratio of six, with the lowest ΔRGB value observed at a PEI/PSMA ratio of 10. The same tendency was observed at all formaldehyde concentrations (0.5, 0.75, 1, 10, and 20 ppm), likely because the response is proportional to the amount of amine groups present at the shell interface in the core–shell nanoparticles. An increase in the amount of amine groups that can react with formaldehyde gas results in a greater change in the pH, which leads to a significant change in the color of the pH indicator dye in the core. As the PEI/PSMA ratio increases from 1 to 4, the thickness of the shell increases and the amount of amine groups is relatively high. However, at higher PEI/PSMA ratios, the thickness of the amine layer decreases owing to the shrinkage of the shell, resulting in low reactivity with formaldehyde gas. Therefore, the most effective detection of aldehyde groups is achieved by the larger amounts of amine group introduced at the PEI/PSMA ratio of four.

Although each sensor exhibited similar tendencies under all formaldehyde conditions, the maximum and minimum ΔRGB values of each dye were different. These differences probably result from the different pH discoloration ranges of the dyes impregnated in the core of the core–shell nanoparticles. Therefore, even if the same amount of amine groups in the shell of each sensor reacts with formaldehyde gas, the ΔRGB value will be different. Among the investigated samples, the MR sensor appeared to have the highest colorimetric response. In contrast, the 4-NP sensor showed the lowest colorimetric response, which is presumably due to a mismatch of the pH range of the dye. Hereafter, this sensor can be used as a MA colorimetric sensor. These results were supported by the morphology analysis (Figure 1 and Figure S1). Thus, both the PEI/PSMA ratio and the pH range of the formaldehyde sensors are important factors for enhancing the sensitivity.

### 3.4. Influence of Various Environmental Factors on the Colorimetric Formaldehyde Sensors

**Figure 5:**
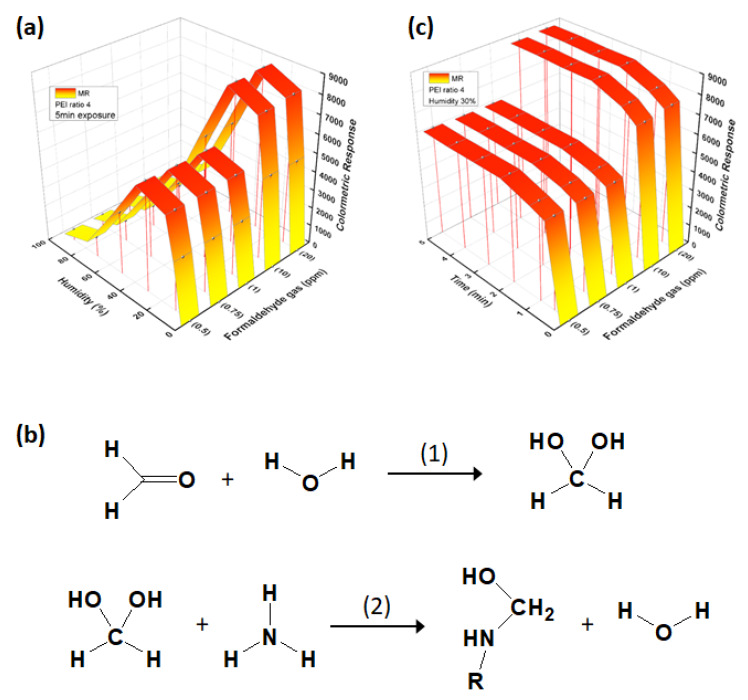
(**a**) Colorimetric response (∆RGB values) of the MR sensor to humidity changes (30–90 RH%). (**b**) Formation mechanism of ethylene glycol via the reaction of formaldehyde with H2O. (**c**) Change in the colorimetric response (∆RGB values) of the MR sensor over time (0–5 min).

To determine the effect of humidity (5, 10, 30, 50, and 90 RH%) and time (0–5 min) on the colorimetric response of the MR sensor, we measured the ΔRGB values, as summarized in Figure 5, Figure S5, Tables S4 and S5. As shown in Figure 5a, the highest colorimetric response was achieved at 30 RH%, whereas the lowest colorimetric response was observed at 90 RH% under all formaldehyde gas conditions. From 10 to 30 RH%, the color change rate increased rapidly, with the rate of increase gradually decreasing at higher values. The observed color changes are affected by Schiff base formation via the nucleophilic addition as well as by protons in water vapor. As the latter effect becomes more prominent as the humidity increases, the color change rate increases.

From 50 RH%, the color change rate decreased sharply and then continued to gradually decrease to 90 RH%. This behavior occurs because the color change is related to the pH change caused by the reaction between the amine groups of the MR colorimetric sensor and formaldehyde.

The reaction between formaldehyde and an amine forms an imine (Schiff base), resulting in a change in pH that causes a change in the color of the pH dye. However, when the humidity increases, instead of the reaction between formaldehyde and the PEI shell layer, formaldehyde reacts with water to form methylene glycol, as shown in Figure 5b. As a result of this continuous reaction, the formation of the Schiff base is reduced and the extent of the pH change is reduced [37]. Subsequent reaction with an amine group of PEI removes methylene glycol, which results in a decrease in the pH and the color change rate. Hence, imine formation cannot occur on the surface of core–shell nanoparticles in the presence of excess humidity. As a result, the MR colorimetric sensor at 30 RH% was more effective for detection of formaldehyde owing to a high extent of imine production. As a result, when using a formaldehyde gas detection sensor at 30 RH%, it is possible to suppress the production of methylene glycol and to observe a distinct color change corresponding to the reaction of PEI and formaldehyde.

The change in the colorimetric response of the MR sensor over time at various formaldehyde gas concentrations is shown in Figure 5c and summarized in Table S4. There is a marked contrast between the responses of the exposed sensors and the initial sensors. Over the first 0.5 min of exposure to formaldehyde gas, the colorimetric response of the sensor rapidly increases. However, as the pH of the MR sensor has already changed, an almost constant response is observed at longer exposure times. Furthermore, similar responses are observed under all formaldehyde conditions. In short, the MR colorimetric sensor exhibits a rapid color change regardless of the concentration of formaldehyde, with a maximum color change rate of 92% observed within 1 min. As a result, small amounts of formaldehyde in the environment can be detected immediately.

## 4. Conclusions

We prepared a colorimetric sensor for formaldehyde based on PSMA/PEI core–shell nanoparticles impregnated with a pH indicator dye. This system exhibited a colorimetric response owing to the reaction between formaldehyde and the amine groups at the shell interface of the core–shell particles. It was found that the PEI/PSMA ratio influenced the structure of the core–shell nanoparticles.

Furthermore, the colorimetric responses of the BCP, MR, and 4-NP sensors varied, indicating that the PEI/PSMA ratio, dye type, and pH range are important factors for improving the sensitivity of the formaldehyde sensor. The unique properties of the dyes were maintained when they were introduced into the core–shell nanoparticles, which is expected to allow a wider range of dyes to be employed in such sensors.

Notably, the colorimetric sensor showed a clear change in color at the lowest formaldehyde concentration (0.5 ppm), confirming its enhanced sensitivity. Advantageously, this sensor provided sensitive and rapid detection (i.e., real-time detection) of formaldehyde. Unlike previous polymer/hybrid nanoparticle-based chemical sensing systems, the developed colorimetric sensors are based on nanoparticles in which both the core and shell are composed of polymers. The findings of this study show that colorimetric sensing based on PCSNPs is a new and promising chemical sensing method. As such sensors have the potential to be easily modified, this approach can be adapted to the sensing of other gases with improved sensitivity and specificity, laying the foundation for future research in various areas, such as accident prevention in chemical industry.

## Figures and Tables

**Table 1:** ΔRGB (%) values of the BCP, MR, and 4-NP sensors with various PEI/PSMA ratios (formaldehyde: 20 ppm).

PEI/PSMA Ratio	Colorimetric Response (ΔRGB)		
	BCP (%)	MR (%)	4-NP (%)
1	5679.24 (66%)	5259.62 (60%)	2557.22 (67%)
2	6571.34 (76%)	6561.02 (75%)	2982.66 (78%)
4	8570.68 (100%)	8718.36 (100%)	3794.50 (100%)
6	4605.40 (53%)	5140.98 (58%)	2143.24 (56%)
8	3568.86 (41%)	4405.06 (50%)	1867.38 (49%)
10	3170.14 (36%)	3670.12 (42%)	1707.64 (45%)

## Supplementary Materials

The following are available online at https://www.mdpi.com/2073-4360/12/5/998/s1, Figure S1: Particle size distribution dependence on PEI/PSMA ratio in PSMA/PEI core–shell nanoparticles, Figure S2: UV-Vis spectra of the pH dyes and the corresponding formaldehyde sensors in *N*-methyl-2-pyrrolidone (NMP): (a) 4-NP, (b) BCP, and (c) MR, Figure S3: ^1^H-NMR spectra (500 MHz) of PEI and PEI/formaldehyde, Figure S4: Colorimetric response (ΔRGB values and difference maps) of (a,b) BCP, (c,d) MR, and (e,d) 4-NP sensors prepared with various PEI/PSMA ratios (0–10) on exposure to 20 ppm formaldehyde gas, Figure S5: Colorimetric response (ΔRGB values) of the MR sensor to temperature changes (30–50 °C), Table S1: Dependence of particle size and shell thickness on PEI content of PSMA/PEI core–shell nanoparticles, Table S2: Colorimetric response of BCP, MR, and 4-NP sensors prepared with various PEI/PSMA ratios (0–10) on exposure to 20 ppm formaldehyde, Table S3: Colorimetric response of BCP, MR, and 4-NP sensors on exposure to 0.5–20 ppm formaldehyde gas (PEI/PSMA ratio of 4), Table S4: Colorimetric response of the MR sensor over time (0–5 min), Table S5: Colorimetric response of the MR sensor to humidity changes (5–90 RH%).
