# Supplementary material for: Colorimetric Visualization Using Polymeric Core–Shell Nanoparticles: Enhanced Sensitivity for Formaldehyde Gas Sensors
Source: Polymers (Basel). 2020 Apr 25;12(5):998. doi: 10.3390/polym12050998 (PMC7285312; doi:10.3390/polym12050998)
Supplement: Supplementary file 1 [file polymers-12-00998-s001.pdf]

Supplementary Information for

Colorimetric Visualization Using Polymeric Core–Shell Nanoparticles: Enhanced Sensitivity for Formaldehyde Gas Sensors

Jae Jung Park[1,2§], Yongsoo Kim[2,3§], Chanmin Lee[2], Jun-Won Kook[2], Donghyun Kim [1],[2],Jung-Hyun Kim[1], Ki-Seob Hwang[2,*], and Jun-Young Lee[2,*]

[1]*Department of Chemical and Biomolecular Engineering, Yonsei University Yonsei-ro 50, Seodaemun-gu, Seoul, 120-749, Korea*

[2]*Research Institute of Sustainable Manufacturing System, Intelligent Sustainable Materials R&D Group, Korea Institute of Industrial Technology, 89 Yangdaegiro-gil, lpjang-myeon, Seobuk-gu, Cheonan-si, Chungcheongnam-do 31056, Korea*

[3]*Department of Polymer Science & Engineering, Sung Kyun Kwan University 2066, Seobu-ro, Jangan-gu, Suwon-si, Gyeonggi-do, Korea*

*To whom correspondence should be addressed, [§]These authors equally contributed.

Tel: +82-41-5898-424 (J.-Y. Lee)

Fax: +82-41-5898-580

E-mail: jaylee@kitech.re.kr (J.-Y. Lee), ks_hwang@kitech.re.kr (K. S. Hwang)

Supplementary Figure. S1.

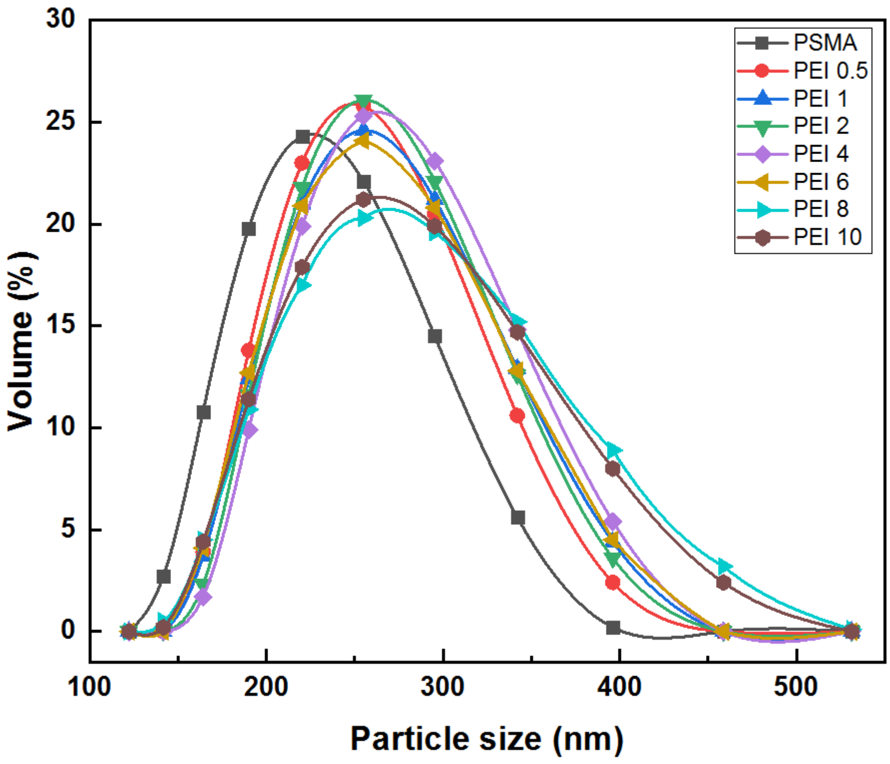

Figure S1. Particle size distribution dependence on PEI ratio in PSMA-PEI core–shell nanoparticles.

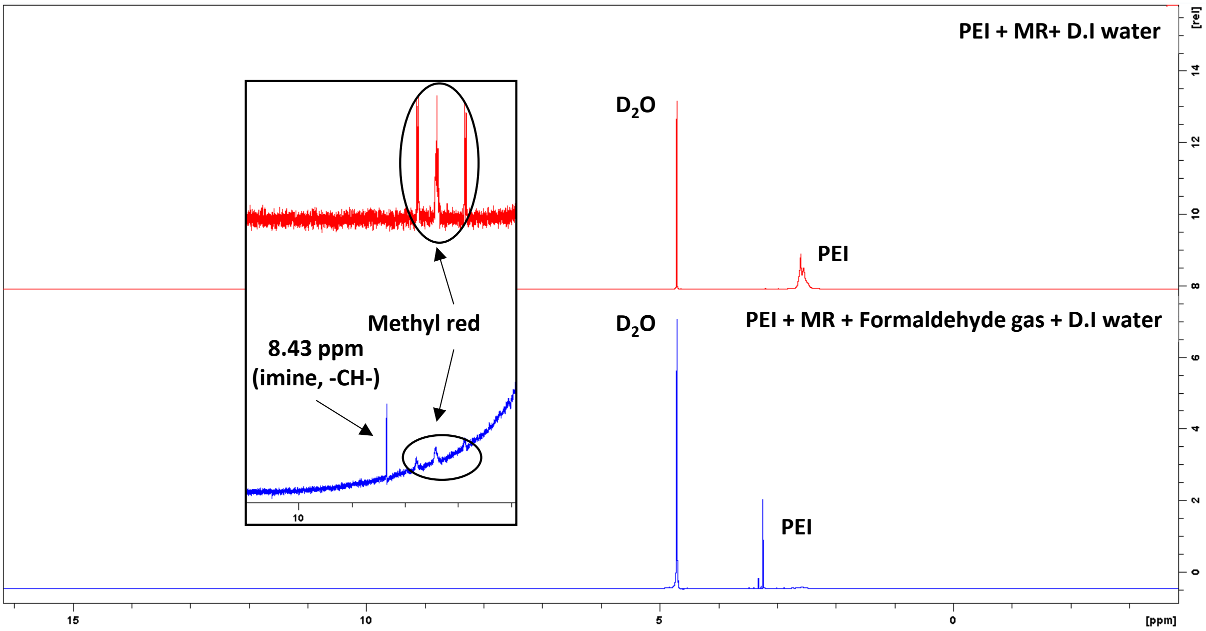

Figure S2. ^{1}H-NMR spectra (500 MHz) of PEI and PEI/formaldehyde.

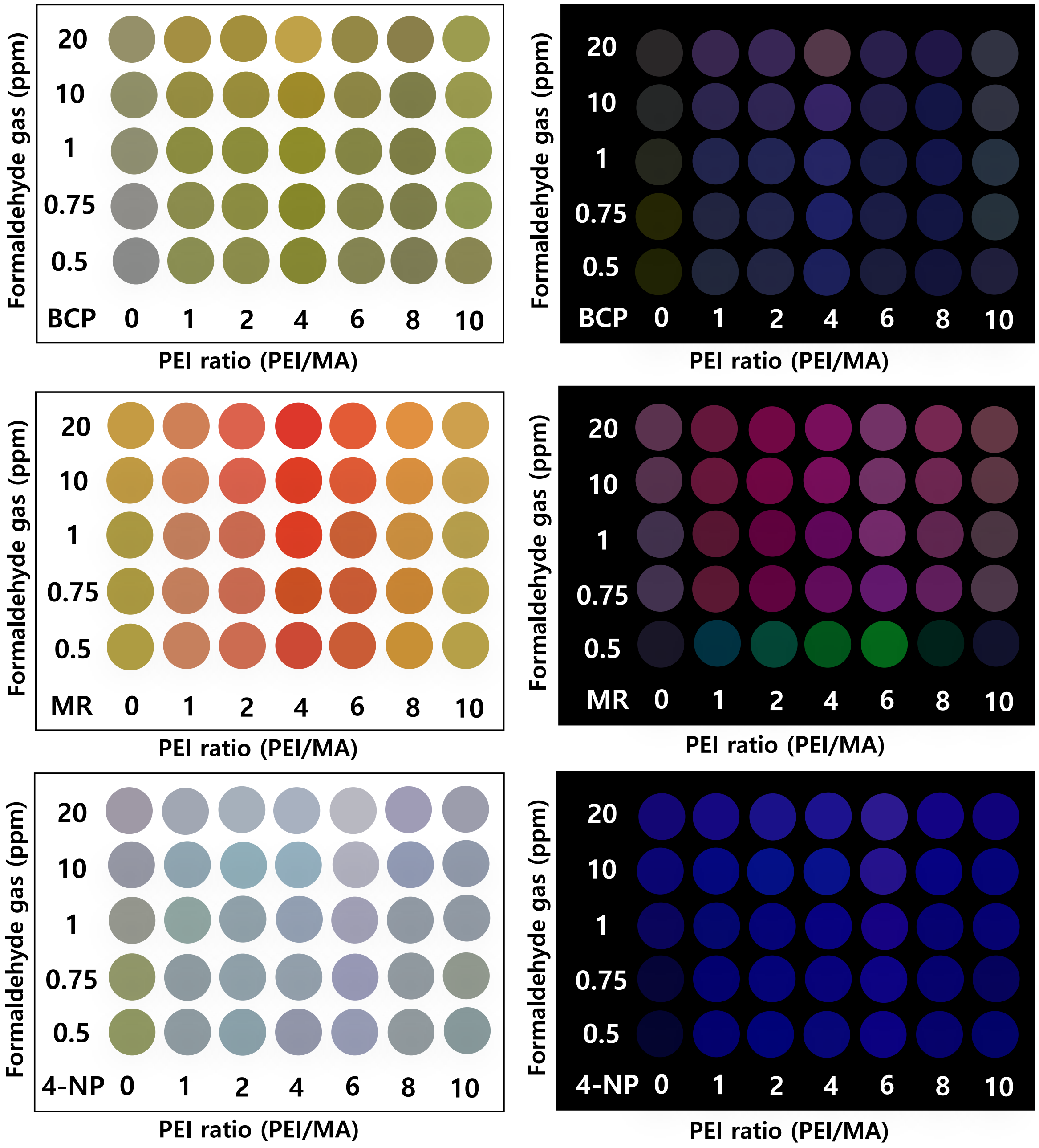

Figure S3. Colorimetric response (RGB values and difference map) of (a,b) BCP, (c,d) MR, and (e,d) 4-NP sensors prepared with various PEI ratios (0–10) on exposure to 20 ppm formaldehyde gas.

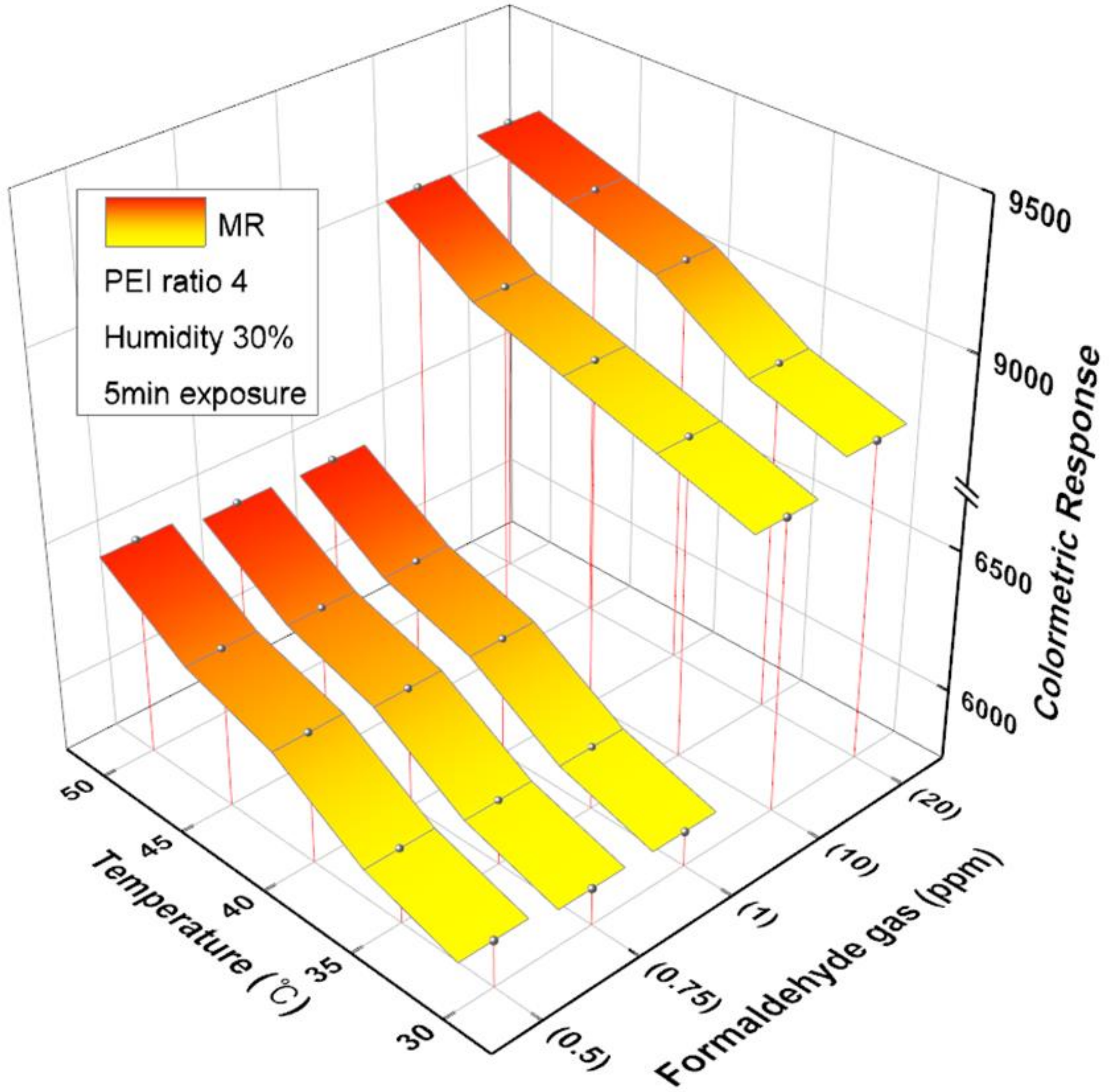

Figure S4. Colorimetric response (△RGB values) of the MR sensor to temperature chang es (30–50 °C).

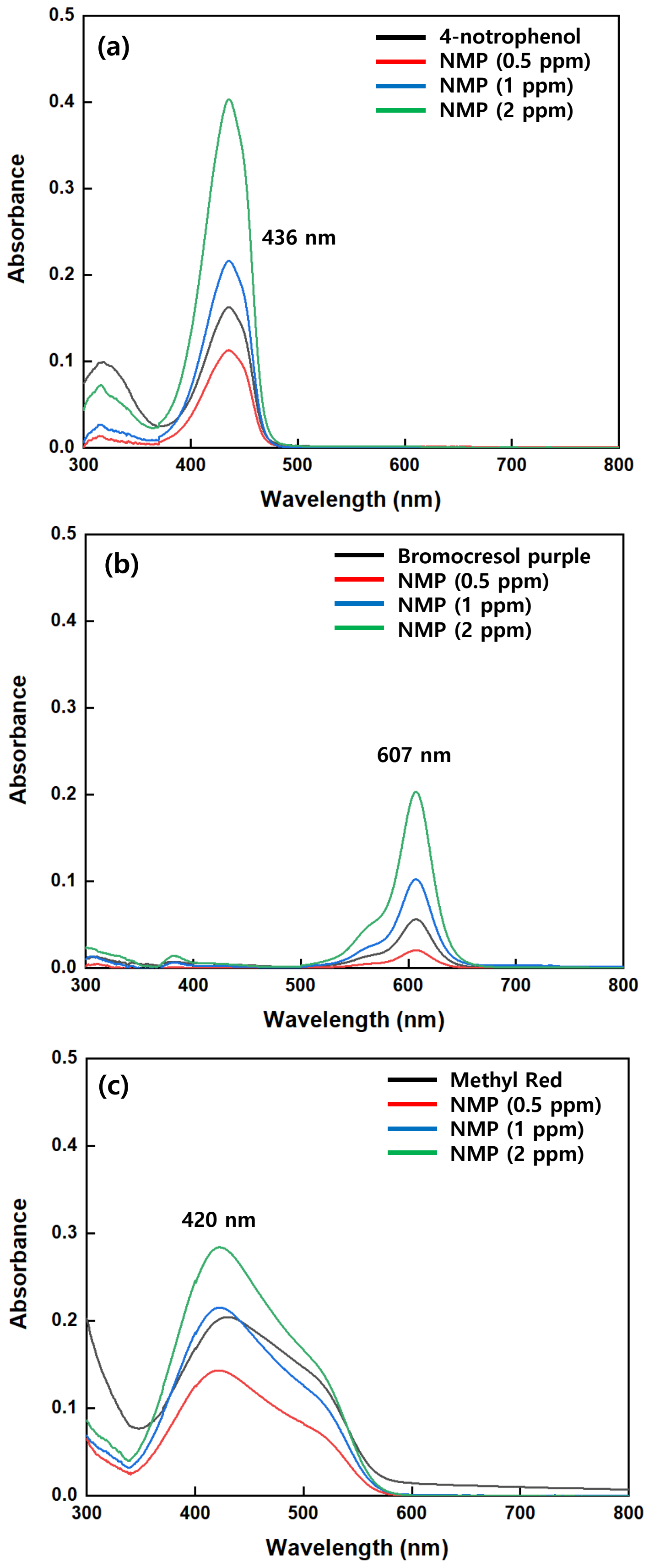

Figure S5. Ultraviolet-Visible Spectrophotometer (UV-Vis) of pH dye (4-NP, BCP and MR) and formaldehyde sensors

Table S1. Dependence of particle size and shell thickness dependence on PEI content of PSMA-PEI core–shell nanoparticles.

PEI ratio	Particle size (nm)	Shell thickness(nm)
0	221	0
0.5	248	27
1	253	32
2	254	33
4	260	39
6	261	40
8	251	30
10	248	27

Table S2. Colorimetric response of BCP, MR, and 4-NP sensors prepared with various PE I ratios (0–10) on exposure to 20 ppm formaldehyde.

PEI ratio	Colorimetric response (ΔRGB)		
	BCP	MR	4-NP
1	5679.24	5259.62	2557.22
2	6571.34	6561.02	2982.66
4	8570.68	8718.36	3794.50
6	4605.40	5140.98	2143.24
8	3568.86	4405.06	1867.38
10	3170.14	3670.12	1707.64

Table S3. Colorimetric response of BCP, MR, and 4-NP sensors on exposure to 0.5–20 ppm formaldehyde gas. (PEI ratio 4)

Formaldehyde gas (ppm)	Colorimetric response (ΔRGB)		
	BCP	MR	4-NP
0.5	5310.34	5916.12	1477.64
0.75	6212.06	5881.22	1584.68
1	6831.88	5895.24	1861.18
10	8313.18	8624.08	3254.98
20	8570.68	8718.36	3794.50

Table S4. Colorimetric response of the MR sensor over time (0.5–5 min).

Time (min)	Formaldehyde (ppm)				
	0.5	0.75	1	10	20
0.5	5037.76	5057.16	5052.63	7330.04	7497.48
1	5342.72	5410.52	5405.84	7934.08	8020.56
2	5846.45	5800.66	5793.41	8523.41	8625.22
3	5856.84	5822.15	5819.15	8537.76	8630.82
4	5861.15	5824.84	5873.54	8608.54	8713.66
5	5916.12	5881.22	5875.24	8624.08	8718.36

Table S5. Colorimetric response of the MR sensor to humidity changes (5–90 RH%).

Humidity (RH%)	Formaldehyde (ppm)				
	0.5	0.75	1	10	20
5	3194.64	3224.55	3231.25	4829.44	4894.90
10	5333.56	5351.71	5405.00	7847.84	8020.56
30	5916.12	5981.22	5985.24	8624.08	8718.36
50	3431.28	3469.79	3548.75	5174.40	5178.62
70	1124.04	1176.20	1175.00	1552.32	1743.6
90	640.76	646.90	705.00	1121.12	1133.34
